# Societal Impacts and Broader Implications of Using Technologies for Caregiving

**DOI:** 10.1093/geroni/igaa057.3039

**Published:** 2020-12-16

**Authors:** Lisa D’Ambrosio, John Rudnik, Joseph Coughlin

**Affiliations:** MIT, Cambridge, Massachusetts, United States

## Abstract

Advances and greater adoption of new technologies may have impacts beyond day-to-day caregiving. This presentation will explore additional implications related to the future of caregiving and technology, including their potential effects on elder abuse, financial fraud, career and labor, social isolation and more. Opinions from the expert panel – which pointed to various possibilities, benefits and challenges – will be shared during this presentation.

